# Breast cancer metastasis to the stomach may mimic primary gastric cancer: report of two cases and review of literature

**DOI:** 10.1186/1477-7819-5-75

**Published:** 2007-07-09

**Authors:** Gregory E Jones, Dirk C Strauss, Matthew J Forshaw, Harriet Deere, Ula Mahedeva, Robert C Mason

**Affiliations:** 1 Department of General Surgery, Guy's and St Thomas' NHS Trust, St Thomas' Hospital, Lambeth Palace Road, London, SE1 7EH, UK; 2 Department of Histopathology, Guy's and St Thomas' NHS Trust, St Thomas' Hospital, Lambeth Palace Road, London, SE1 7EH, UK

## Abstract

**Background:**

The stomach is an infrequent site of breast cancer metastasis. It may prove very difficult to distinguish a breast cancer metastasis to the stomach from a primary gastric cancer on the basis of clinical, endoscopic, radiological and histopathological features. It is important to make this distinction as the basis of treatment for breast cancer metastasis to the stomach is usually with systemic therapies rather than surgery.

**Case presentations:**

The first patient, a 51 year old woman, developed an apparently localised signet-ring gastric adenocarcinoma 3 years after treatment for lobular breast cancer with no clinical evidence of recurrence. Initial gastric biopsies were negative for both oestrogen and progesterone receptors. Histopathology after a D2 total gastrectomy was reported as T4 N3 Mx. Immunohistochemistry for Gross Cystic Disease Fluid Protein was positive, suggesting metastatic breast cancer. The second patient, a 61 year old woman, developed a proximal gastric signet-ring adenocarcinoma 14 years after initial treatment for breast cancer which had subsequently recurred with bony and pleural metastases. In this case, initial gastric biopsies were positive for both oestrogen and progesterone receptors; subsequent investigations revealed widespread metastases and surgery was avoided.

**Conclusion:**

In patients with a history of breast cancer, a high index of suspicion for potential breast cancer metastasis to the stomach should be maintained when new gastrointestinal symptoms develop or an apparent primary gastric cancer is diagnosed. Complete histopathological and immunohistochemical analysis of the gastric biopsies and comparison with the original breast cancer pathology is important.

## Background

Metastatic spread to the upper gastrointestinal tract is infrequently reported with cancers of the breast, lung, kidney and malignant melanoma [1]. The most common sites of breast cancer metastasis are the skeleton, lungs and the liver; the stomach, peritoneum, colon, retroperitoneum and the small bowel have all been reported as potential sites of metastatic involvement [2]. Metastatic spread to the stomach may occur many years after the initial treatment for breast cancer. It may prove very difficult to distinguish from a primary gastric cancer on clinical, endoscopic, radiological and histopathological features [3-5]. However, it is important to make this distinction as the basis of treatment for breast cancer metastasis to the stomach is usually with systemic therapies rather than surgery [6,7].

The authors present two cases of breast cancer metastasis to the stomach, both of which were initially considered to represent primary gastric cancer. The authors also review current literature with a particular emphasis on the utility of immunohistochemistry in differentiating between primary gastric cancer and gastric metastasis from breast cancer.

## Case Presentations

### Patient 1

A fifty-one year old woman presented with weight loss and vague epigastric pain. Three years previously, she had undergone a wide local excision and axillary dissection for a right sided breast lump. Postoperative histology showed a completely excised grade II invasive carcinoma (T2) with associated intermediate grade DCIS. On immunohistochemistry, the tumour was found to be positive for both oestrogen and progesterone receptors but negative for E-cadherin, suggesting a predominantly lobular tumour type; all eight excised lymph nodes were clear of metastases (N0). She had subsequently received adjuvant radiotherapy to the breast. As she was unable to tolerate either tamoxifen or anastrozole due to side effects (persistent hot flushes), surgical oophorectomy had been performed. She required tibolone to treat menopausal symptoms. She had been regularly reviewed in breast clinic without any clinical evidence of recurrence.

**Figure 1:**
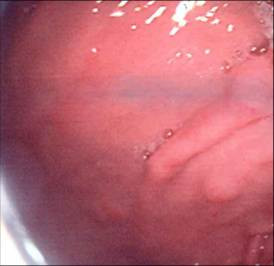
Endoscopic view of antral polyps in patient 1, biopsies of which confirmed signet-ring adenocarcinoma.

**Figure 2:**
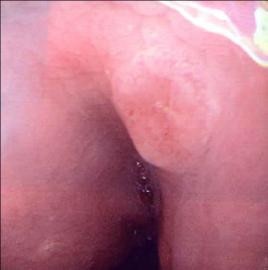
Endoscopic view of fundal polyp in patient 1, biopsies of which confirmed signet-ring adenocarcinoma.

She was initially investigated with an upper gastrointestinal endoscopy. This demonstrated only small antral polyps, which were biopsied (Figure 1). Histology revealed gastric mucosa infiltrated by poorly differentiated adenocarcinoma of a signet-ring pattern, with immunostaining negative for oestrogen, progesterone receptors and Her-2. Due to the unexpected nature of these findings, a further upper gastrointestinal endoscopy was performed with repeat biopsies from both proximal and distal stomach which, again, were consistent with signet-ring type adenocarcinoma (Figure 2). Computed tomography (CT) could not define a definite primary gastric cancer; there was no evidence of lymph node or metastatic spread (Tx N0 M0).

**Figure 3:**
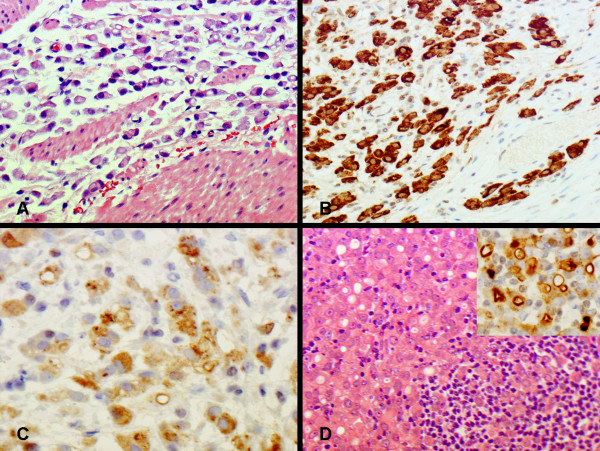
An invasive adenocarcinoma is present in the gastrectomy specimen from patient 1. Numerous signet ring cells are seen in the gastric wall (panel A). Carcinoma cells are immunohistochemically positive for CK7 (panel B) and gross cystic disease fluid protein (GCDFP) (panel C). Metastatic carcinoma cells in the lymph node (panel D) are also positive for GCDFP (insert).

The patient was counselled for radical curative surgery in view of an apparent localised primary gastric cancer. A D2 total gastrectomy with Roux-en-Y reconstruction was performed and the patient made an uneventful postoperative recovery. Post operative histology revealed a poorly differentiated adenocarcinoma of the stomach, invading into the duodenum, with 40 out of 41 lymph nodes involved (pT4 N3 Mx). Immunohistochemistry performed on the stomach and lymph nodes was positive for CK7 and GCDFP (Gross Cystic Disease Fluid Protein) (Figure 3) and negative for CK20, E-cadherin and Her-2. The tumour in the lymph nodes, but not the stomach, was strongly positive for both oestrogen and progesterone receptors. Consequently, the diagnosis was revised to metastatic breast cancer. The patient was considered for further hormonal therapy. Subsequently she developed widespread bony metastases. She is currently receiving bisphosphonates and palliative care.

### Patient 2

A sixty-one year old woman had undergone a mastectomy and axillary dissection for a left sided breast cancer fourteen years previously. Postoperative histology revealed a grade III 4 × 2 × 2 cm infiltrating lobular carcinoma; the tumour was positive for both oestrogen and progesterone receptors but negative for E-cadherin. Fourteen out of fifteen sampled lymph nodes contained metastases. The patient then received adjuvant chemotherapy, radiotherapy to the breast and five years' treatment with tamoxifen. She remained disease free on follow up until one year ago when she was diagnosed with bony metastases and a malignant pleural effusion. This was treated with paclitaxel chemotherapy, followed by letrozole and ibandronate, with symptomatic improvement and apparent disease remission.

**Figure 4:**
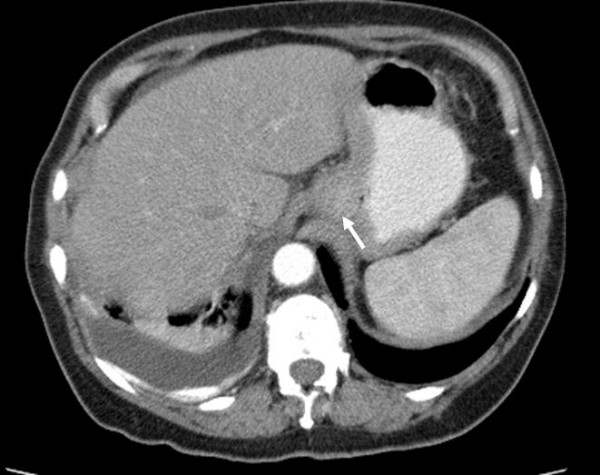
CT abdomen with oral contrast in patient 2 demonstrating thickening below the oesophagogastric junction (indicated by arrow) and residual right sided pleural effusion.

Nine months after this recurrence she presented with progressive dysphagia and weight loss. An upper gastrointestinal endoscopy revealed a tumour arising from the gastro-oesophageal junction and occupying the lesser curve of the anterior gastric wall. Biopsies revealed a poorly differentiated adenocarcinoma of signet ring cell type. CT showed an extensive gastric cancer just below the oesophagogastric junction without any obvious lymph node or metastatic spread; this was staged as T3 N0 M0 (Figure 4).

**Figure 5:**
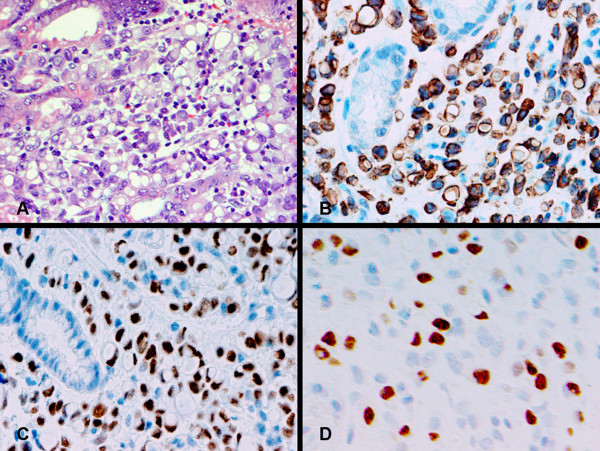
Gastric biopsy from patient 2 is infiltrated by a poorly differentiated adenocarcinoma with signet ring cell morphology (panel A). Immunohistochemistry showing positive staining for CK7 (panel B), oestrogen receptor (panel C) and progesterone receptor (panel D).

She was referred to our unit for a surgical opinion. She was planned for a staging laparoscopy but, before this could be performed, she developed a left sided upward gaze. CT scan of the brain showed a left retro-orbital space occupying lesion consistent with a metastasis. Immunohistochemistry on the biopsies from the stomach became available at this stage and were positive for both oestrogen and progesterone receptors (CK7, ER and PR positive; CK20 and Her-2 negative), consistent with metastatic breast carcinoma (Figure 5). Further surgical intervention was avoided and the patient has received further chemotherapy and radiotherapy to her brain.

## Discussion

Both of the patients in this report were initially diagnosed with an apparent primary gastric cancer. Ultimately this diagnosis was revised to metastatic breast cancer, but only after surgery had been performed in patient 1. The incidence of breast cancer metastasis to the stomach in long term follow up and post mortem studies has been estimated at 2–18% [3,6,8-11]. This may occur many years after the diagnosis of the primary breast lesion and, in 90 – 94% of patients, there will be concurrent sites of breast cancer metastasis [6,7]. Patient 2 had been treated for recurrent breast cancer prior to her presentation with a gastric tumour.

The clinical presentation of a breast cancer metastasis to the stomach is often indistinguishable from primary gastric cancer, as seen in the two patients in this report. Common symptoms include dyspepsia, anorexia, epigastric pain, early satiety, vomiting and bleeding. The most common pattern of breast cancer metastasis to the stomach is a linitis plastica with diffuse infiltration of the submucosa and muscularis propria; less commonly, discrete nodules or external compression may occur [7]. Madeya and Borsch reported that 73% of patients with gastric metastases had diffuse intramural infiltration imitating linitis plastica [12]. Diffuse infiltration of the stomach is characteristic of metastases from invasive lobular carcinoma: Taal et al reported that 83% of patients with gastric metastasis had lobular breast carcinoma as the primary pathology [6]. Patient 1 had a linitis plastica type pattern whereas patient 2 had a discrete proximal gastric lesion.

A high index of suspicion for metastatic breast cancer should be maintained when a patient develops gastric pathology with a history of breast cancer. Endoscopic, radiological and histological evaluation is essential to discriminate between primary gastric cancer and breast cancer metastasis to the stomach. Macroscopic endoscopic findings are usually unhelpful in identifying the underlying pathology. Since metastatic gastric infiltration is frequently limited to the submucosa and seromuscular layer, endoscopic evaluation may be normal in 50% of cases or only show discrete mucosal abnormalities indistinguishable from other tumours or benign disease [13]. Radiological findings on computed tomography or barium studies include encasement of the whole stomach as seen in linitis plastica, multiple lesions of the stomach or extrinsic lesions of the gastric wall [4]. Deep and extensive biopsies should be performed at endoscopy. Their histology should be compared with the primary breast cancer pathology as the histologic picture may be similar. However, lobular carcinoma of the breast may produce a signet ring morphology which can be confused with a primary signet ring or diffuse-type gastric adenocarcinoma [7]. Both of the patients in this study had been previously treated for lobular breast cancer.

Detailed immunohistochemical analysis may be the only consistent method for differentiating between metastatic and primary gastric carcinoma. Although oestrogen and progesterone receptor positivity in the gastric biopsies suggest breast cancer metastasis to the stomach, it is worth noting that oestrogen and progesterone receptor positivity have been reported in 32% and 12% of patients with primary gastric cancer [14]. However, these findings are based upon studies using first-generation antibodies against Oestrogen receptor β (ERβ) which are no longer used in standard practice. Taal et al investigated whether immunohistochemical detection with second-generation antibodies against Oestrogen receptor α (ERα) can be used to diagnose gastric metastasis of breast carcinoma [15]. In their study none of the primary gastric carcinomas expressed ERα. Moreover, no cases with an ER- primary breast carcinoma and an ERα+ carcinoma in a gastric biopsy specimen were found. Therefore they concluded that ERα expression can be reliably used to diagnose gastric metastasis of breast carcinoma. They also investigated if the expression pattern of E-cadherin could be of help in the differential diagnosis of primary gastric cancer versus metastatic breast carcinoma. In their study absence of E-cadherin staining was significantly related to metastatic breast carcinoma. It appears that the absence of E-cadherin expression in an adenocarcinoma in a gastric biopsy should raise the possibility of metastatic breast carcinoma and ERα positivity can be reliably used to diagnose gastric metastasis of breast carcinoma.

The absence of positive oestrogen and progesterone receptors in patient 1's gastric biopsies led to the initial assumption that this was a primary gastric cancer. Positive monoclonal staining with GCDFP-15 (gross cystic disease fluid protein-15) has been found to be a sensitive (55–76%) and specific (95–100%) marker to correctly identify a malignant lesion as metastatic breast carcinoma [5,16-21]. This marker is a monoclonal antibody of gross cystic disease fluid protein-15 (GCDFP-15) which is detected in macroscopic breast cyst fluid and in the plasma of patients with breast cancer [22,23]. There is an excellent correlation between GCDFP-15 positivity and the origin of a metastatic breast adenocarcinoma [17]. Wick demonstrated reactivity for GCDFP-15 in 76 of 105 breast carcinomas (72%) [16]. The diagnosis of metastatic breast cancer in patient 1 was eventually confirmed on the basis of GCDFP immunohistochemistry.

In common with other sites of metastatic breast cancer, breast cancer metastasis to the stomach should be treated systemically [7]. The choice of systemic treatment is based upon presenting symptoms, age, general performance status, receptor status and previous systemic treatments. The response rate to chemotherapy and hormonal therapy varies: the median survival in two small series of patients receiving systemic treatment for breast cancer metastasis to the stomach and gastrointestinal tract varied between 10 and 28 months [6,24]. In a series of 51 patients with gastric metastases from the breast, hormonal therapy (27%) was performed almost as frequently as chemotherapy (33%) [7]. The options for hormonal therapy included tamoxifen, oophorectomy or progesterone as first-line treatment, and aminogluthetimide, androgens or prednisolone as second line treatment. New anti-hormonal drugs also include aromatase inhibitors and GNRH analogues in pre-menopausal patients. Although combination cyclophosphamide, methotrexate and 5-fluorouracil (CMF) tends not to be used in the metastatic setting, new anti-neoplastic drugs are now available and include taxanes, capecitabine and trastzumab if c-erbB2 is positive.

One study has shown that a partial remission with a clear palliative effect can be obtained in only 46% of patients receiving systemic therapy with no obvious difference in response rates between hormonal treatment and chemotherapy. It is worth noting that symptomatic treatments alone such as acid reduction could only be performed in 20% of the patients in this study due to their poor general condition or extensive prior treatment [7]. McLemore et al reported a median overall survival after diagnosis of 28 months in 73 patients with breast cancer metastasis to the gastrointestinal tract [24]: whilst advanced age at diagnosis and gastric metastasis had a negative effect on survival, treatment with systemic chemotherapy or tamoxifen had a positive effect on survival. It is worth noting that there is a low response rate to chemotherapy in invasive lobular carcinoma patients which must be must taken into consideration when choosing the most appropriate treatment [25].

The complications of breast cancer metastasis to the stomach can be managed in a similar manner to primary gastric cancer: endoluminal stents can be used for gastric outlet obstruction; bleeding may be controlled by endoscopic or endovascular therapy [26-31]. Although surgical resection has been considered in selected patients, the role of surgery is usually limited as the gastric metastasis reflects systemic disease [6,32]. Palliative surgery has not been shown to affect overall survival [24]. Surgery should be limited to palliative bypass in those patients where less invasive measures fail to palliate their gastric outlet obstruction.

## Conclusion

In patients with a history of breast cancer, a high index of suspicion for potential breast cancer metastasis to the stomach should be maintained when new gastrointestinal symptoms develop or an apparent primary gastric cancer is diagnosed. Complete histopathological and immunohistochemical analysis of the gastric biopsies and comparison with the original breast carcinoma pathology is essential to support the diagnosis of metastatic breast carcinoma. Appropriate systemic treatment for metastatic breast carcinoma is the preferred treatment.

## Competing interests

The author(s) declare that they have no competing interests.

## Authors' contributions

GE Jones Collected data and wrote "Case presentations" section.

DC Strauss Researched the literature and wrote "Discussion" section.

MJ Forshaw Edited and revised the manuscript.

H Deere Provided histopathological diagnoses and revised the manuscript.

U Mahadeva Provided histopathological diagnoses and revised the manuscript.

RC Mason Consultant responsible for the care of the patients and is the guarantor of the manuscript.

All authors have read and approved the manuscript.
